# Interfacial Characteristics of a Fly Ash-Based Artificial Aggregate

**DOI:** 10.3390/ma19132886

**Published:** 2026-07-06

**Authors:** Xiaoxing Zeng, Qijun Yu, Jiangxiong Wei, Fang Zhang, Qian Sun

**Affiliations:** 1 School of Materials Science and Engineering, South China University of Technology, Guangzhou 510640, China; mide_zxxing@aliyun.com (X.Z.); jxwei@scut.edu.cn (J.W.); 15866507231@163.com (Q.S.); 2 University International College, Macau University of Science and Technology, Macau 999078, China

**Keywords:** fly ash, artificial aggregate, alkali activation, interface transition zone, strength, elastic modulus

## Abstract

A fly ash-based artificial aggregate with a compressive strength of >60 MPa was prepared via cement activation and alkali activation using >75% fly ash as the principal raw material. The mechanical properties of concrete prepared using this aggregate and the characteristics of the interfacial transition zone (ITZ) were compared with those of concrete containing natural aggregate. The results indicated that the compressive strength of concrete prepared using artificial aggregate was lower than that of concrete prepared using natural aggregate by about 19.0–27.6%. Scanning electron microscopy (SEM) revealed that the cement paste bonded tightly to the surface of the natural aggregate; the width of ITZ was 20–30 µm. The ITZ between the cement paste and the fly ash-based artificial aggregate exhibited a relatively loose structure at 28 d, with a width of 30–40 µm; however, the ITZ became narrower and denser at 90 d. EDS indicated that the principal hydration products were calcite crystals and C-S-H gel in the ITZ of natural aggregate concrete and artificial aggregate concrete. According to nanoindentation tests, for both cement pastes with natural and artificial aggregates, the elastic modulus of the ITZ at 28 d was >10 GPa, and it increased slightly at 90 d. The ITZ between the alkali-activated paste and limestone exhibited a relatively dense structure, with a width of 20–30 µm. The ITZ between the alkali-activated paste and the fly ash-based artificial aggregate exhibited a relatively loose structure with numerous pores at 28 d and had a width of 30–40 µm; however, the ITZ became narrower and denser at 90 d. The principal hydration products were N-A-S-H and C-A-S-H in the two kinds of aggregate concrete. Whether the alkali-activated paste contained natural aggregate or artificial aggregate, the elastic modulus of the ITZ at 28 d was 5–6 GPa, and it increased rapidly to >10 GPa by 90 d. The performance of ITZ is primarily influenced by the matrix materials, while also being influenced by aggregates and curing conditions. Qualitative and quantitative analyses revealed the formation mechanisms of artificial and natural aggregates in different matrices. Through continuous hydration and polymerization reactions, artificial aggregates gradually form narrower and denser interfacial transition zones with different matrices, especially in alkali-activated matrices. The continuously improved performance of the ITZ makes it less prone to forming cracks between the ITZ and the artificial aggregate. This study provides an important theoretical basis for the application of fly ash-based artificial aggregates, which can also be used to produce high-strength concrete.

## 1. Introduction

Concrete is one of the most extensively used building materials in construction projects. Specifically, aggregates, including sand and stone, account for 70% of the volume of concrete, and the global annual demand for concrete aggregates has exceeded 40 billion tons [1]. The consumption of sand and stone aggregates in China is particularly high, accounting for 40% of global consumption. The annual consumption of sand and stone aggregates in China is estimated at 16–22 billion tons [2]. Recently, natural aggregates such as pebbles and river sand have largely been depleted, leading to the extensive exploitation of quarry resources such as granite and limestone for use as coarse and fine aggregates in concrete. As finite natural resources, quarry materials should be conserved and their extraction restricted based on sustainable development principles. To ensure the sustainable development of resources, the identification of alternative materials for concrete aggregates has become an important development direction in the construction industry. Current approaches include the replacement of natural aggregates with recycled aggregates or the production of artificial aggregates [3,4]. Specifically, the preparation of artificial aggregates using solid wastes offers significant application potential and environmental benefits [5,6,7,8]. Recent studies have highlighted the potential of alternative cementitious systems to enhance not only material properties but also environmental outcomes, including waste valorization and a reduced carbon footprint. For instance, eco-friendly reinforced concrete elements incorporating alternative aggregates have demonstrated viable performance under extreme conditions such as fire exposure [9]. Such findings underscore the importance of linking microstructural evolution with broader sustainability goals, including resource efficiency and design practice.

In concrete samples, a zone with structural and property differences from the bulk paste forms between the aggregate particle surface and the surrounding cement paste owing to factors such as aggregate surface roughness and the water–cement ratio. This region is referred to as the interfacial transition zone (ITZ). The ITZ constitutes the region of paste–aggregate composite interaction, and its properties are critical to the overall mechanical performance and durability of concrete. This region is typically considered the weakest zone in concrete. Numerous studies have demonstrated that the relatively poor mechanical properties of the cement–concrete ITZ are mainly attributed to low density, high porosity, microcracks, and high Ca(OH)_2_ concentration [10,11].

The elemental distribution within the ITZ, its variation along different directions, and its thickness are all related to the type of aggregate used [12]. A previous study reported that sandstone and limestone aggregates adsorbed abundant ions but also released ions that underwent chemical reactions at the aggregate surfaces, thereby enhancing the microhardness of the ITZ. However, similar reactions might not occur in basalt, granite, or marble [13]. Furthermore, for crushed natural aggregates, the thickness of the ITZ increases with increasing coarse aggregate size, while the mechanical performance of the concrete declines accordingly [14,15].

Recycled and artificial aggregates have been widely applied in recent years. These materials exhibit structural characteristics that differ from those of natural aggregates, thus influencing the properties of composite materials such as concrete. For example, recycled aggregates typically contain multiple constituent materials, and concrete incorporating recycled aggregates tends to exhibit a coarser aggregate–paste ITZ than conventional concrete [16]. Compared with the cement paste–aggregate ITZ in ordinary concrete, the ITZ in concrete incorporating recycled aggregates is generally wider, and the microhardness of both the aggregate and the cement matrix on either side of the ITZ is lower [17,18]. In solid waste-based artificial aggregate concrete, the artificial aggregate typically constitutes the weakest component, resulting in a higher cracking rate than that observed in concrete containing natural aggregates. In concrete with natural aggregates, cracks typically initiate within the ITZ and the cement matrix [19,20]. A previous study showed that, in microhardness tests, the comparatively weak interfacial transition zone (ITZ) was observed in natural aggregate concrete rather than in geopolymer aggregate concrete. The micro-level observations demonstrated the existence of dense microstructures in geopolymer aggregate/matrix interfacial regions, especially when the water–binder ratio is low [4]. Several studies have shown that the interfacial properties of alkali-activated concrete are superior to those of conventional cement concrete [21,22,23].

The type, morphology, and preparation method of aggregates may all affect the characteristics of the concrete ITZ. Most natural crushed stone aggregates are relatively inert and do not directly react with cementitious materials such as cement. In contrast, the surfaces of fly ash-based artificial aggregates contain abundant partially reacted and unreacted fly ash particles, which confer potential reactivity. Currently, there are still many gaps in the understanding of the interfacial transition zone characteristics of fly ash-based artificial aggregates, such as the following aspects: (1) the existence and formation mechanism of the ITZ are controversial, (2) the dynamic interface evolution law brought about by potential active aggregates is unclear, (3) the differences between the interfacial zones of different cementitious material matrices and fly ash-based artificial aggregates are not yet clear, and (4) the evolution process of the mechanical properties in the interfacial transition zone of fly ash-based artificial aggregates and the fracture propagation path are unclear. Unlike natural aggregates, the “potentially active aggregate” characteristic of fly ash-based artificial aggregates brings about entirely new interface science issues—the ITZ is no longer a simple physical bonding interface but a dynamic reaction zone of chemical-physical coupling. This characteristic presents both challenges and opportunities: if the aggregate-paste interface reaction can be actively controlled from the source of material design, it is hoped that the bottleneck of the weak zone of the ITZ in traditional concrete can be broken through, and a true “synergistic strengthening of the entire system” can be achieved. In this study, both natural and artificial aggregates were crushed via the same method, and coarse aggregates with the same grading were prepared. The characteristics of the ITZ between different aggregates and cement paste, as well as between different aggregates and alkali-activated fly ash paste, were compared by using SEM, EDS, and nanoindentation tests. The findings offer an important basis for further investigation into the applications of fly ash-based artificial aggregates.

## 2. Materials and Methods

### 2.1. Raw Materials

**Figure 1:**
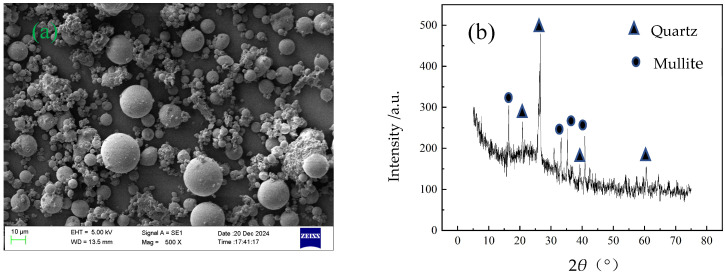
SEM morphology and XRD patterns of fly ash (**a**) SEM morphology, and (**b**) XRD patterns.

Ordinary Portland cement (OPC) of strength grade 52.5 was used (Macau, China), and the slag powder was S95-grade ground granulated blast-furnace slag (GGBS) (Guangzhou, China). The fly ash (FA) was Class II ash (Hongkong, China), produced by CLP Power Hong Kong Limited, with a density of 2.60 g/cm^3^, and the Blaine specific surface area of 3400 cm^2^/g, the FA particles predominantly ranged from 1.28 to 73.33 μm, with a median diameter (D50) of 15.07 μm, the SEM and XRD test results are given in Figure 1. The chemical composition of all materials, as determined by the suppliers in accordance with EN 196-2:2013 [24], is summarized in Table 1. The granite coarse aggregate exhibited a particle size of 5–10 mm and an apparent density of 2620 kg/m^3^. The limestone coarse aggregate exhibited a particle size of 5–10 mm and an apparent density of 2610 kg/m^3^ (Jiangmen, China). SKY8588 was utilized as a high-efficiency polycarboxylate superplasticizer (Hongkong, China). The alkali activator comprised sodium silicate (industrial grade) with a modulus of 3.28 and a solid content of 36.73%. The Na_2_O content was 8.79%, and the SiO_2_ content was 27.94% (Foshan, China). Analytically pure sodium hydroxide was used to adjust the modulus of the alkali activator to 1.2 (98.5%, Guangzhou, China), after which the solution was allowed to stand for 24 h. BaCl_2_ was applied as a retarder for the alkali-activated materials (99.5%, Guangzhou, China).

### 2.2. Preparation of Fly Ash-Based Artificial Aggregates and Concrete Without Fine Aggregate

#### 2.2.1. Preparation of Fly Ash-Based Artificial Aggregates

Cement–fly ash artificial aggregate paste and alkali-activated fly ash aggregate paste were prepared according to previously reported mixing ratios (Table 2 and Table 3). Afterward, the prepared pastes were poured into triple test molds and cured in a standard curing chamber. After 24 h, the molds were removed, and the specimens were transferred to a temperature-and humidity-controlled curing chamber and cured further for 24 h at 75 °C and a relative humidity of 100%. Some studies have shown that high-temperature curing can enhance the strength development of alkali-activated materials, with a suitable temperature range of 60–80 °C [25,26,27]. Subsequently, the samples were collected and crushed using a laboratory PE60 × 100 jaw crusher (Wuxi Jianyi Instrument & Mechinery Co., Ltd., Wuxi, China). The crushing stages were divided into two levels: 0–5 mm and 5–10 mm, with a recovery rate of 20–25% for particles with a diameter of 5 mm to 10 mm. Particles measuring 5–10 mm were selected for use as coarse artificial aggregates. The bulk density of the cement-activated fly ash aggregate was 1000 g/L, the apparent density was 1950 kg/m^3^, the 24-h water absorption rate was 13.5%, and the porosity was 11.6%. The bulk density of the alkali-activated fly ash aggregate was 925 g/L, the apparent density was 1910 kg/m^3^, the 24-h water absorption rate was 14.0%, and the porosity was 20.7%.

#### 2.2.2. Preparation of Concrete Without Fine Aggregate

Different types of cement-based concrete without fine aggregate and alkali-activated concrete were prepared according to previously reported mixing ratios (Table 4 and Table 5). The cementitious materials and natural aggregates were mixed at a mass ratio of 1:1. The amount of artificial aggregate used was calculated based on the same volume as that of the natural aggregate. According to Chinese Standard JC/T 958-2005 [28]: Flow table for determining mortar fluidity, the spread was controlled to 180–220 mm. Afterward, the prepared mixtures were poured into triple cement mortar test molds and cured in a standard curing chamber. The samples were demolded after 24 h. Some samples were then cured under standard conditions, at 20 ± 2 °C and a relative humidity greater than 95%, for different ages, while others were cured at 75 °C and a relative humidity of 100% for 24 h. Mechanical properties were tested at the end of high-temperature curing, while the remaining samples were placed in a standard curing room for testing at different curing ages. Samples at 28 d were cut and prepared for analysis of interfacial properties. In the sample designations, GA denotes granite aggregate, LA denotes limestone aggregate, CA denotes cement–fly ash aggregate, and AA denotes alkali-activated fly ash aggregate.

### 2.3. Test Methods

#### 2.3.1. Mechanical Property Testing of Aggregates and Concrete Without Fine Aggregate

Natural aggregates were processed into 40 × 40 × 160 mm prismoids to ensure geometrical consistency with the cement–fly ash aggregates and the alkali-activated fly ash aggregates. The mechanical properties of the different types of aggregates and concrete without fine aggregate were determined with reference to the ISO standard method for strength testing of cement mortar. The compressive strength and flexural strength of the concrete without fine aggregate were measured at standard curing ages of 3 d, 7 d, 28 d, and 56 d. For flexural strength, three samples were used, and the calculation formula is Rf = 1.5FL/b^3^ (L = 100 mm, b = 40 mm). Individual values exceeding the average by ±10% should be discarded. The compressive strength was calculated using the arithmetic mean of six valid specimens, accurate to 0.1 MPa, with a permissible coefficient of variation of ≤3%.

#### 2.3.2. Microstructural Testing

The microstructure of concrete without fine aggregate was examined using scanning electron microscope (SEM, Zeiss EVO 18, Carl Zeiss AG, Oberkochen, Germany), with an accelerating voltage of 0.2–30 kV, a working chamber of 365 mm (Φ) × 275 mm (h), and a maximum sample height of 145 mm. Its five-quadrant backscattered electron detector (BSE) enhances low-voltage performance and provides morphological information, and the samples were prepared with a gold-sprayed conductive coating. Specimens were analyzed at curing ages of 28 d and 90 d (56 d of standard curing followed by 24 d in a curing chamber at 20 °C and 50% relative humidity). The specific sample preparation method used in this study is as follows: The 40 × 40 × 160 mm prismoid specimen was cut into 40 × 40 × 5 mm specimens, which were then divided into 10 × 10 × 5 mm samples and encapsulated in epoxy resin. After the epoxy resin was completely cured, the specimen was sanded and polished. The obtained specimens were immersed in anhydrous ethanol for 24 h to prevent further hydration and then dried in an oven at 60 °C to a constant weight. After vacuum drying, the sample was surface-sputtered with gold. Under a vacuum of 10^−1^ to 10^−2^ Torr, metal atoms are sputtered through glow discharge to uniformly cover the sample surface with a 5–10 nm metal film. Particular attention was given to the width, principal products, and morphology of the ITZ between different gel materials and different types of aggregate.

#### 2.3.3. Micro-Mechanical Testing of the Interface

**Figure 2:**
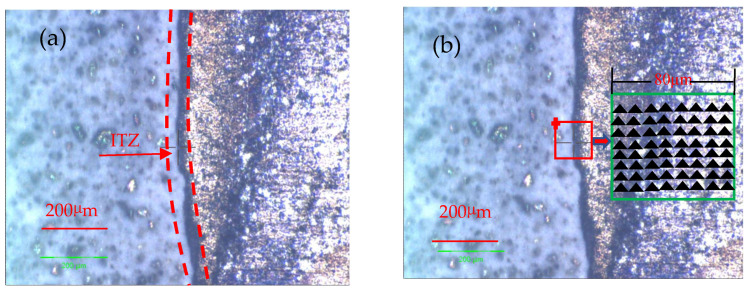
Microscopic images of ITZ and schematic illustration of the indented area: (**a**) Microscopic images of ITZ, (**b**) Schematic illustration of the indented area.

Micro-mechanical testing of the interface used the nanoindenter (TTX-NHT3, Anton Paar GmbH, Graz, Austria). Specimens of cement concrete without fine aggregate and alkali-activated concrete were prepared as in Section 2.3.2 before testing. For each concrete group (fly ash aggregate concrete and natural aggregate concrete), three representative sample cross-sections were selected. The instrument’s built-in microscope is used to locate the interfacial transition zone, and nanoindentation testing is performed starting from the paste or aggregate side. Microscopic images of the ITZ and a schematic illustration of the indented area are shown in Figure 2. During nanoindentation testing, the maximum load was 5 mN, and both the loading and unloading rates were 5 mN/min. The peak load was maintained for 5 s. After the test was completed, data statistics were performed separately for the three areas: matrix, interface transition zone, and aggregate. More than 20 valid data points were collected for each area.

## 3. Results and Discussion

### 3.1. Basic Properties of Different Types of Aggregate

**Figure 3:**
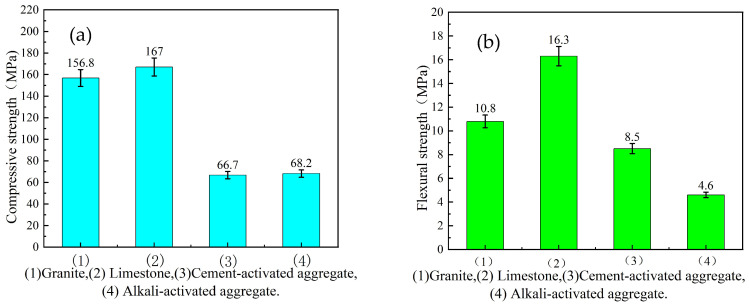
Comparison of mechanical properties of various aggregates: (**a**) Compressive Strength, (**b**) Flexural strength.

The mechanical properties of the different types of aggregate were compared (Figure 3). Notably, the compressive strengths of the crushed granite and limestone both exceeded 150 MPa. The compressive strength of the artificial aggregates was significantly lower than that of the natural aggregates. Specifically, the average compressive strength of the cement-activated fly ash-based artificial aggregate reached 66.7 MPa, while that of the alkali-activated fly ash-based artificial aggregate reached 68.2 MPa. The flexural strength of the natural aggregates was >10 MPa, with limestone exhibiting the highest value (16.3 MPa). The flexural strength of the cement-activated fly ash-based aggregate was 8.5 MPa, which was similar to that of granite. In contrast, the flexural strength of the alkali-activated fly ash-based aggregate was only 4.6 MPa, which indicated relatively low toughness.

**Figure 4:**
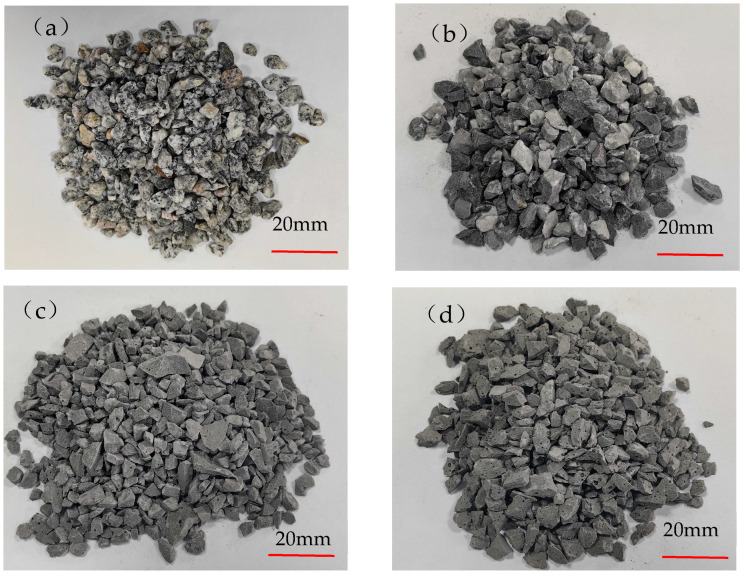
Comparison of mechanical properties of various aggregates: (**a**) Granite, (**b**) Limestone, (**c**) Cement-activated aggregate, (**d**) Alkali-activated aggregate.

Figure 4 shows the appearance of the crushed fly ash-based artificial aggregate and the different types of natural aggregate. Notably, the fly ash-based artificial aggregates are predominantly grey, and their edges and corners are similar to those of ordinary crushed natural aggregates. The artificial aggregates exhibited prominent edges and corners, which contributed to the mechanical strength of the aggregate ‘skeleton’ formed within the concrete. Regarding the surface morphology of the artificial aggregates, numerous pores were observed on the surface, particularly in the alkali-activated fly ash-based aggregates. The pore structure of the artificial aggregates was mainly affected by material properties. For example, in a highly alkaline environment, residual ammonium salts in fly ash (such as NH_4_HSO_4_ and (NH_4_)_2_SO_4_) undergo a strong chemical reaction, rapidly converting into free ammonia (NH_3_) and releasing it. The volatilization of this gas leaves numerous interconnected pores within the hardened slurry, significantly increasing its porosity. The ammonium ion content in the fly ash was 553 mg/kg, tested according to Chinese standard GB/T 39701-2020 [29], and the standard stipulates that the limit for ammonia content is 210 mg/kg. Studies have shown that a high ammonium ion content in fly ash increases the air content and reduces the bulk density of concrete [30,31]. The paste of cement-activated fly ash-based materials exhibited good fluidity owing to the longer setting time, allowing entrapped bubbles to dissipate gradually. In contrast, the paste of alkali-activated fly ash-based materials exhibited a relatively high bubble content, as the shorter setting time hindered bubble dissipation. Consequently, the water absorption of the artificial aggregates was relatively high.

### 3.2. Mechanical Properties of Cement Concrete Without Fine Aggregate and Alkali-Activated Concrete

**Figure 5:**
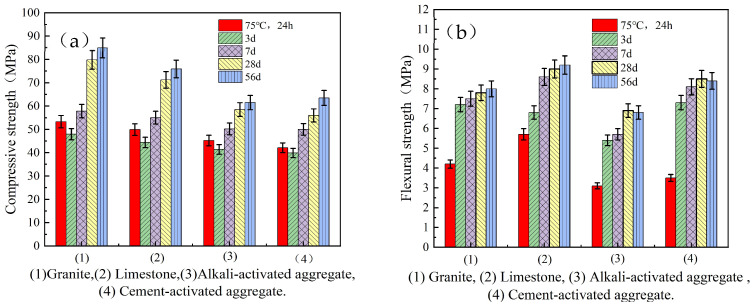
Comparison of the mechanical properties of cement concrete without fine aggregate: (**a**) Compressive Strength, (**b**) Flexural strength.

The mechanical properties of cement concrete without fine aggregate were compared (Figure 5). The compressive strength value of specimens cured for 24 h at high temperature (75 °C) was between those of specimens subjected to standard curing for 3 d and 7 d. High-temperature curing had a limited effect on the strength of cement-based gel materials. Under standard curing conditions, the compressive strength of concrete without fine aggregate increased progressively with curing age. The highest compressive strength at 56 d occurred in concrete with granite but no fine aggregate, reaching 84.9 MPa. The compressive strengths of concretes incorporating cement-activated artificial aggregates and alkali-activated artificial aggregates at 56 d were similar (63.5 MPa and 61.5 MPa, respectively). The strength of concrete prepared using artificial aggregates was 27.6% lower than that of concrete containing natural aggregates. Furthermore, the flexural strength value of natural aggregate concrete cured for 24 h at high temperature (75 °C) was between those of samples subjected to standard curing for 3 d and 7 d. The flexural strength of artificial aggregate concrete after high-temperature curing was lower than that of specimens subjected to standard curing for 3 d. This finding indicated that high temperature might facilitate the formation of excessive calcium hydroxide (Ca[OH]_2_) crystals or low-density C-S-H gel. Consequently, additional microcracks could develop within the ITZ between the aggregate and the cement paste, weakening interfacial bonding and thereby reducing the flexural strength of the materials. Among the different types of aggregate, the concrete containing limestone aggregate exhibited the highest flexural strength, reaching 9.2 MPa at 56 d.

The flexural strengths of artificial aggregate with granite and cement-activated artificial aggregate were comparable (8.0 MPa and 8.4 MPa, respectively). The lowest flexural strength was observed in alkali-activated artificial aggregate, with a value of 5.8 MPa. Thus, for the same gel systems, the flexural strength of concrete was related to aggregate characteristics. Generally, the paste–aggregate ITZ is the region with the weakest mechanical properties in concrete, and it exhibits the greatest influence on flexural strength. The flexural strength of alkali-activated artificial aggregate was substantially lower than that of cement-activated artificial aggregate. The cement content in cement-activated aggregate was relatively low, and FA constituted the principal component. The pH of the hardened cement–fly ash body was lower than that of fresh cement paste. When fresh cement paste came into contact with the cement-activated aggregate, the high-alkaline environment of the paste further activated the partially reacted FA within the cement, thereby enhancing the bonding strength of the ITZ. In contrast, a different reaction was observed at the ITZ formed between fresh cement paste and alkali-activated artificial aggregate. The alkali-activated aggregate was a relatively weak component owing to its low fracture resistance. In addition, numerous visible pores were observed on the aggregate surfaces, which ultimately weakened interfacial performance. Consequently, the toughness of concrete containing alkali-activated FA aggregate was reduced.

**Figure 6:**
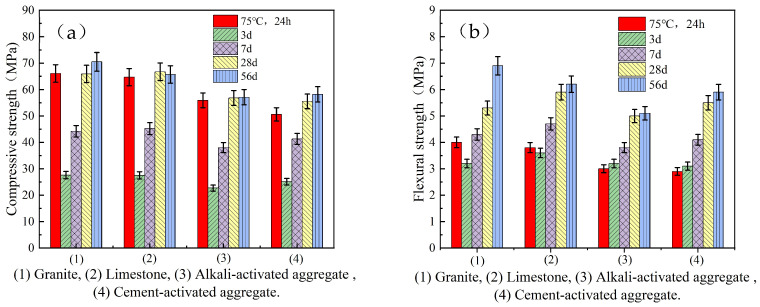
Comparison of the mechanical properties of alkali-activated concrete without fine aggregate: (**a**) Compressive Strength, (**b**) Flexural strength.

The mechanical properties of alkali-activated concretes without fine aggregate were compared (Figure 6). The compressive strength of samples cured for 24 h at high temperature (75 °C) was similar to that of samples subjected to standard curing for 28 d. This finding indicated that high-temperature curing effectively promoted the strength development of alkali-activated materials [32,33]. Under standard curing conditions, the compressive strength of alkali-activated concrete without fine aggregate increased progressively with curing age, although the rate of increase became relatively low after 28 d. The compressive strength of alkali-activated concrete containing granite without fine aggregate reached the highest value (70.5 MPa) at 56 d, while those of concretes incorporating cement-activated artificial aggregate and alkali-activated artificial aggregate at 56 d were similar (58.2 MPa and 57.1 MPa, respectively). The compressive strength of concrete prepared using artificial aggregates was 19.0% lower than that of concrete prepared using natural aggregates. The flexural strength values of natural aggregate samples cured for 24 h at high temperature (75 °C) were between those of samples subjected to standard curing for 3 d and 7 d. The flexural strength of specimens containing artificial aggregates after high-temperature curing was lower than that of specimens subjected to standard curing for 3 d. The highest flexural strength for alkali-activated concrete was observed in the mix containing granite aggregates, ultimately reaching 6.9 MPa. The flexural strengths of mixes containing limestone aggregate and cement-activated artificial aggregate were comparable (6.2 MPa and 5.9 MPa, respectively). The lowest flexural strength occurred in the mix containing alkali-activated artificial aggregate, with a value of 5.1 MPa.

Although fly ash-based artificial aggregates can be used to prepare high-strength concrete, such as concrete with a strength exceeding 70 MPa, the failure mechanism is still relatively complex. Hence, they are not currently suitable for use in structural concrete, but are acceptable for low- to medium-strength or non-structural applications (e.g., lightweight fill, insulation blocks).

### 3.3. Morphological Analysis of the Interfacial Transition Zone (ITZ) for Cement Concrete

#### 3.3.1. Characteristics of the ITZ Between Cement Paste and Natural Aggregate

**Figure 7:**
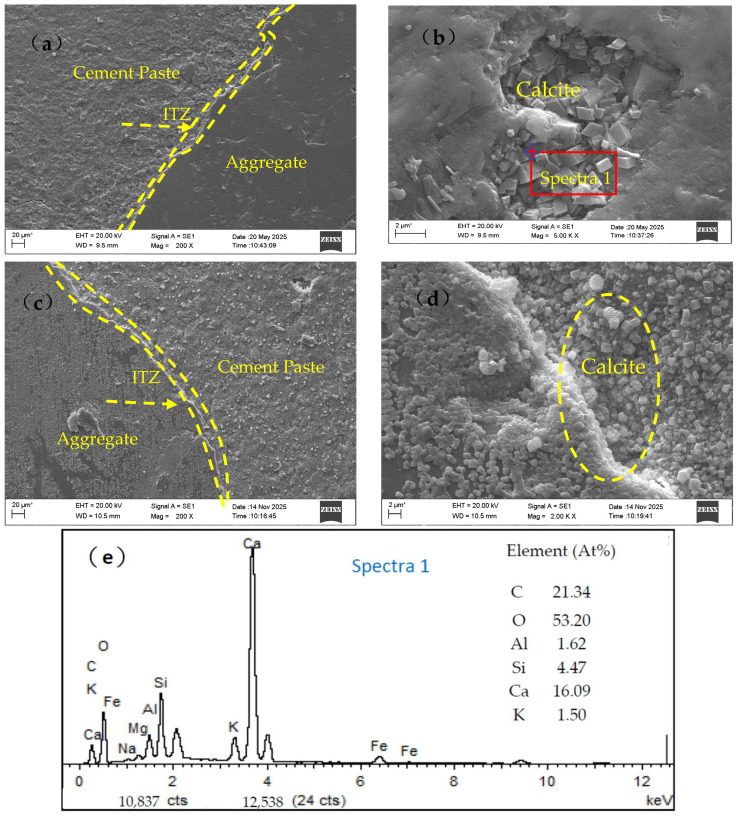
Morphology of the ITZ between cement paste and granite aggregate and the principal hydration products: (**a**) ITZ width at 28 d, (**b**) Main hydration products of ITZ at 28 d, (**c**) ITZ width at 90 d, (**d**) Main hydration products of ITZ at 90 d, (**e**) EDS of ITZ at 28 d.

Figure 7 shows the morphology of the ITZ between cement paste and natural aggregates (e.g., granite and limestone), as well as the surrounding hydration products. Notably, the cement paste bonded tightly with both granite and limestone, resulting in distinct ITZ boundaries. The ITZ width was 20–30 µm, and the principal hydration products were square-shaped calcite crystals and C-S-H gel. The calcite was formed by carbonation. EDS testing showed that the Ca/Si ratio in ITZ was 3.6, which indicates a high CH crystal content and a low degree of C-S-H gel polymerization. A substantial amount of calcite filled the pores within the ITZ, and its accumulation increased porosity, thereby weakening ITZ performance. The morphology of the ITZ at 28 d and 90 d changed slightly. After 90 d, the hydration products within the ITZ became denser, although the region remained susceptible to crack development.

#### 3.3.2. Characteristics of the ITZ Between Cement Paste and Artificial Aggregate

**Figure 8:**
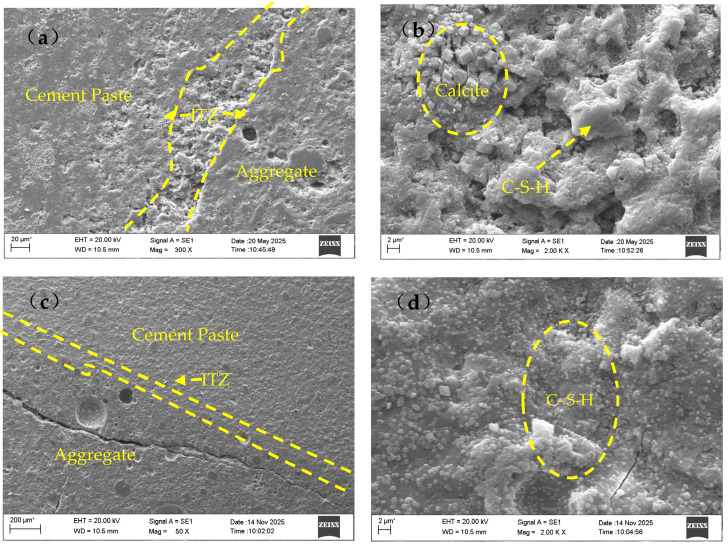
Morphology of the ITZ between cement paste and alkali-activated artificial aggregate and the principal hydration products: (**a**) ITZ width at 28 d, (**b**) Main hydration products of ITZ at 28 d, (**c**) ITZ width at 90 d, (**d**) Main hydration products of ITZ at 90 d.

**Figure 9:**
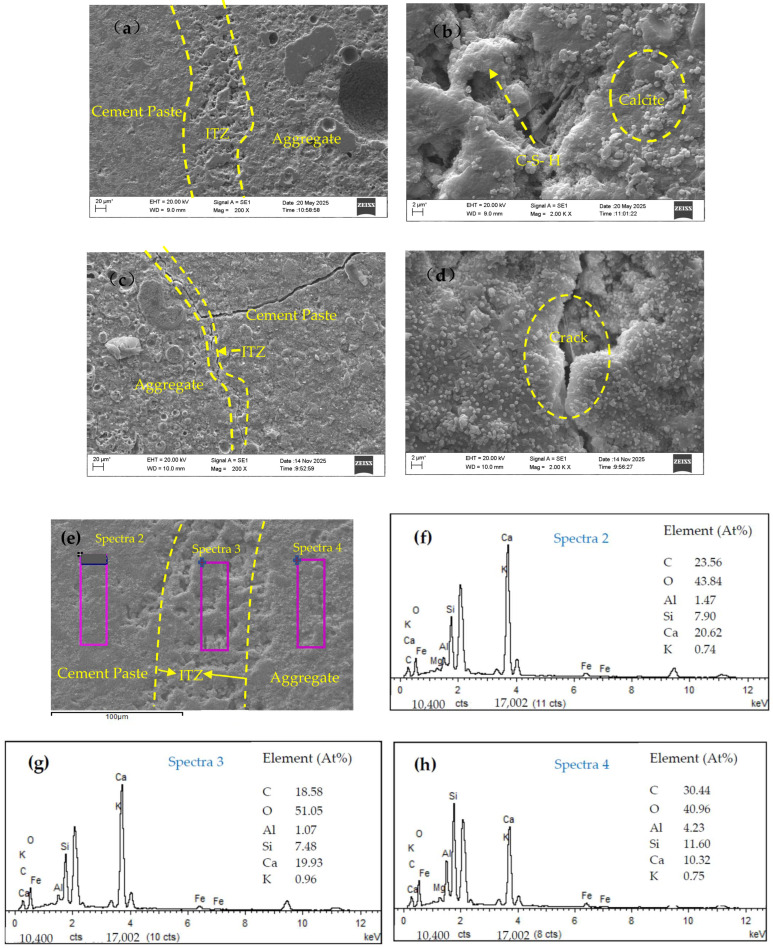
Morphology of the ITZ between cement paste and cement-activated artificial aggregate and the principal hydration products: (**a**) ITZ width at 28 d, (**b**) Main hydration products of ITZ at 28 d, (**c**) ITZ width at 90 d, (**d**) Main hydration products of ITZ at 90 d, (**e**) EDS line scanning of the ITZ, (**f**) Spectra 2, (**g**) Spectra 3, (**h**) Spectra 4.

Figure 8 and Figure 9 show the morphology of the ITZ between cement paste and artificial aggregate, as well as the principal hydration products. Notably, the ITZ between the cement paste and the alkali-activated artificial aggregate at 28 d was relatively distinct (Figure 8). It exhibited a relatively loose structure, and the products on both sides displayed similar morphologies. The ITZ width was 30–40 µm. EDS tests showed that Ca/Si in the cement paste and ITZ were 2.61 and 2.66, respectively, and Ca/Si for cement-activated aggregate was 0.89 (Figure 9). This indicates that the main products in the ITZ are similar to the products in the cement paste. At 90 d, the ITZ between the cement paste and the artificial aggregate became substantially denser, with no distinctly defined boundaries. This finding revealed that chemical reactions occurred continuously within the ITZ under suitable conditions, thereby enhancing its properties. Square-shaped calcite crystals and amorphous C- S-H gel were the principal hydration products in the ITZ between the cement paste and the alkali-activated aggregate. Furthermore, the ITZ between the cement paste and the cement-activated aggregate at 28 d exhibited a relatively loose structure, which became denser at 90 d, although cracks developed between the paste and the aggregate (Figure 9). This observation was attributed to the differing shrinkage characteristics of the cement paste and the cement-activated aggregate.

### 3.4. Morphological Analysis of the Interfacial Transition Zone (ITZ) for Alkali-Activated Concrete

#### 3.4.1. Characteristics of the ITZ Between Alkali-Activated Paste and Natural Aggregate

**Figure 10:**
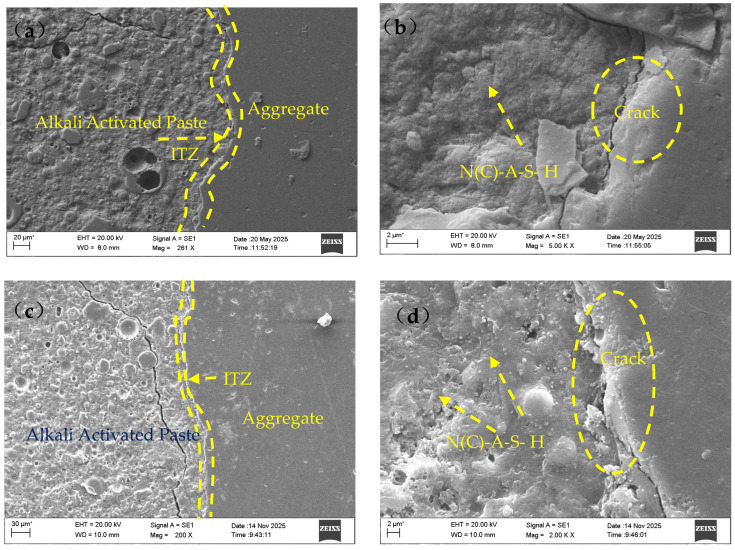
Morphology of ITZ between alkali-activated paste and granite aggregate and main hydration products: (**a**) ITZ width at 28 d, (**b**) Main hydration products of ITZ at 28 d, (**c**) ITZ width at 90 d, (**d**) Main hydration products of ITZ at 90 d.

Figure 10 shows the morphology of the ITZ between alkali-activated paste and natural aggregate, as well as the principal hydration products. Notably, the ITZ between the alkali-activated paste and granite aggregate was clearly defined and exhibited tight bonding, mainly due to physical bonding forces. Furthermore, the ITZ exhibited a relatively dense structure, with a width range of 20–30 µm.

**Figure 11:**
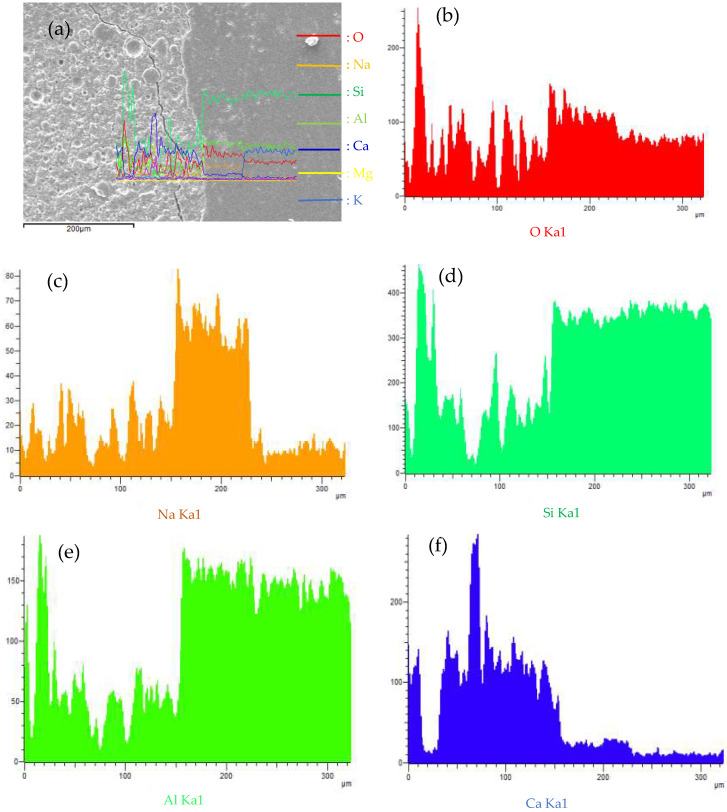
EDS line scan of the ITZ between alkali-activated paste and granite aggregate: (**a**) Distribution of elements, (**b**) O Ka1, (**c**) Na Ka1, (**d**) Si Ka1, (**e**) Al Ka1, (**f**) Ca Ka1.

As shown in Figure 11, the boundary between aggregate and ITZ can be distinctly identified according to the element distributions. Si and Al are the dominant elements in rock, which are far less abundant in the alkali-activated fly ash geopolymer. The distribution of Al, Si, Ca, and Na in the paste indicates the presence of C-A-S-H and N-A-S-H gels. Microcracks, associated with the high shrinkage of alkali-activated materials, were observed in the ITZ adjacent to the aggregate [34,35,36]. The morphology of the ITZ at 28 d and 90 d changed only slightly, and microcracks remained. Cracks within the alkali-activated paste were wider than those in the ITZ. So the ITZ is not the weakest region in AAFS concrete due to its desired micromechanical properties and compact microstructure compared to the paste matrix [23].

#### 3.4.2. Characteristics of the ITZ Between Alkali-Activated Paste and Artificial Aggregate

**Figure 12:**
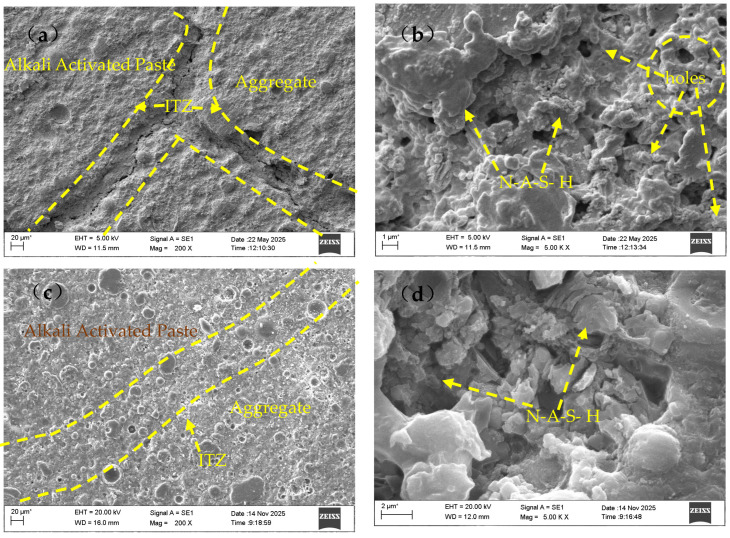
Morphology of ITZ between alkali-activated paste and alkali-activated artificial aggregate and main hydration products: (**a**) ITZ width at 28 d, (**b**) Main hydration products of ITZ at 28 d, (**c**) ITZ width at 90 d, (**d**) Main hydration products of ITZ at 90 d.

**Figure 13:**
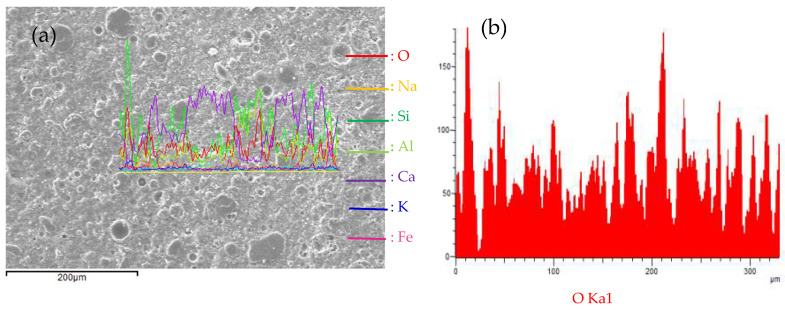

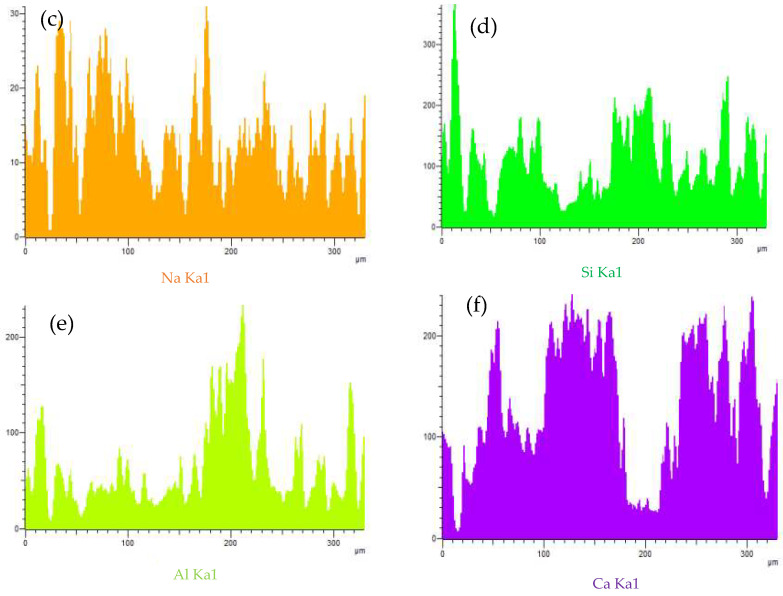
EDS line scan of the ITZ between alkali-activated paste and alkali-activated paste aggregate: (**a**) Distribution of elements, (**b**) O Ka1, (**c**) Na Ka1, (**d**) Si Ka1, (**e**) Al Ka1, (**f**) Ca Ka1.

Figure 12 shows the morphology of the ITZ between the alkali-activated paste and alkali-activated artificial aggregate, the principal hydration product was N-A-S-H gel. Notably, the morphology of the ITZ between alkali-activated paste and alkali-activated aggregate changed significantly with curing age. At 28 d, the ITZ between the newly formed paste and the existing aggregate was susceptible to crack and pore development. The structures on both sides of the ITZ were similar, and the ITZ width was 30–40 µm. At 90 d, the ITZ became narrower and narrower, even difficult to distinguish. Specifically, the alkali-activated paste essentially integrated with the alkali-activated aggregate, indicating that continuous reactions occurred between the alkali-activated materials and the artificial aggregate, thereby increasing the compactness of the ITZ. It can be explained by the continued reaction of the outer layer of artificial aggregate with the preexisting matrix, which results in less porosity and orientation deposition of crystals [37]. Qian found that concrete prepared with polymer aggregates (paste compressive strength over 140 MPa) had higher strength than concrete prepared with natural aggregates, and the micro-level observations demonstrated the existence of dense microstructures in higher strength polymer aggregates [4]. In Figure 13, Na and Al show distinct enrichment within the ITZ, indicating the presence of N-A-S-H gel, and Ca is relatively scarce in the interfacial transition region, indicating a lower concentration of C-A-S-H gel in this area. During the early stages of reaction between the alkali-activated paste and the alkali-activated aggregate, the hydration products within the ITZ exhibited relatively loose structures owing to the high water–binder ratio, combined with numerous nanopores. With increasing curing age, the ITZ became denser. 

**Figure 14:**
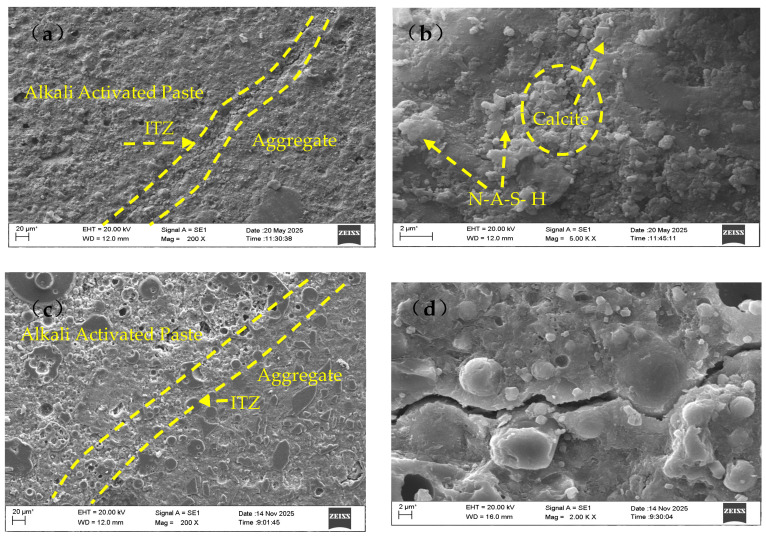
Morphology of ITZ between alkali-activated paste and cement-activated artificial aggregate and main hydration products: (**a**) ITZ width at 28 d, (**b**) Main hydration products of ITZ at 28 d, (**c**) ITZ width at 90 d, (**d**) Main hydration products of ITZ at 90 d.

Figure 14 shows the morphology of the ITZ between the alkali-activated paste and cement-activated artificial aggregate. The formation and development of the ITZ are similar to those of alkali-activated fly ash aggregate. The principal hydration products in the ITZ between the alkali-activated paste and the cement-activated aggregate included N-A-S-H and substantial amounts of square-shaped CaCO_3_ crystals. The internal structure of the gels was relatively dense, although cracks were present within the ITZ. These cracks might be attributed to differences in shrinkage characteristics between materials of different properties.

### 3.5. Mechanical Properties of the ITZ in Cement and Alkali-Activated Concrete

The elastic modulus of cement hydration products is a key mechanical parameter that reflects the resistance of these products to elastic deformation. The principal hydration products of cement include tobermorite (C-S-H gel) and calcium hydroxide (Ca[OH]_2_), while alkali-activated materials also contain gels such as N-A-S-H. The microstructure and properties of these phases play an important role in the overall performance of cement-based materials. Typically, the elastic modulus of silicate gel, C-S-H, and CH is 30–50 GPa, 10–30 GPa, and 30–40 GPa, respectively. The elastic modulus in pore regions is generally <10 GPa [38,39].

Table 6 shows the statistical analysis of the elastic modulus of ITZ for different types of concrete. Notably, the elastic modulus of cement paste ranges from 22–26 GPa and increases gradually with curing age. On the other hand, whether cement paste with natural aggregate or cement paste with artificial aggregate, the elastic modulus of ITZ at 28 d was >10 GPa, but less than half the amount of cement paste; this was attributed to the principal product and the relatively loose, porous structure of the ITZ, inferred via scanning electron microscopy (Section 3.3). The elastic modulus of ITZ increased slightly at 90 d, it is mainly due to the continuous reaction of cement and the artificial aggregate, which underwent secondary reactions with Ca(OH)_2_ in the cement paste, leading to the formation of additional hydration products, thereby increasing the compactness of the ITZ and enhancing its properties. In summary, cement paste and the ITZ in cement paste are non-homogeneous materials, which are multiphase composite materials mainly consisting of C-S-H gel, unhydrated calcium hydroxide cement particles, and pores. Therefore, in terms of micromechanical property testing, the standard deviation of nanoindentation test datas for these materials will be very large. The standard deviation of the ITZ region is typically larger than that of the cement paste, stemming from the unique structural gradient superimposed on its heterogeneity.

The elastic modulus of the alkali-activated paste is about 20 GPa, which is lower than that of cement paste, and it gradually increases with curing age. Notably, whether alkali-activated paste with natural aggregate or alkali-activated paste with artificial aggregate, the elastic modulus of ITZ at 28 d was 5–6 GPa, which is only a quarter of that of alkali-activated paste, but it increased to more than 10 GPa at 90 d, indicating ongoing reactions within the ITZ. This process improved ITZ’s performance. The elastic modulus of the ITZ at 28 d was <10 GPa. This observation was attributed to the relatively high water–gel ratio and the accumulation of hydration products within the ITZ owing to the high surface roughness and abundant surface pores of the artificial aggregate. The increased water–gel ratio contributed to the lower strength of the alkali-activated FA polymer. At 90 d, the elastic modulus of the ITZ increased significantly, approaching that of the alkali-activated materials. This trend indicated that the products within the ITZ underwent continuous polymerization and hydration reactions, thereby increasing ITZ density. Thus, alkali-activated materials improved the properties of the ITZ. Specifically, artificial aggregates with intrinsic reactivity did not align with the ‘weak interface’ model of conventional concrete. A denser and mechanically improved ITZ formed between alkali-activated materials and fly ash-based artificial aggregates, which is different from traditional cement-based materials. The underlying mechanism was attributed to the continuous growth of the gel network resulting from chemical compatibility and the mechanical interlocking effect provided by the porous aggregate surface.

In summary, the performance of ITZ is primarily influenced by the matrix materials, while also being influenced by aggregates and curing conditions. Although the early performance of fly ash-based artificial aggregates in the ITZ with various matrices is inferior to that of natural aggregates, fly ash-based artificial aggregates owing to their inherent potential reactivity, can progressively optimize the performance of the ITZ through later-stage physical and chemical reactions.

## 4. Conclusions

In this study, by comparing fly ash-based artificial aggregates with natural aggregates, the effects of different aggregates on the mechanical properties and interfacial characteristics of concrete are studied to improve the performance of fly ash-based artificial aggregates. The conclusions obtained are drawn as follows:

(1) Fly ash-based artificial aggregates with compressive strengths > 60 MPa were prepared via cement activation and alkali activation, using >75% FA as the principal raw material. Cement concrete with a compressive strength of 77.2 MPa and alkali-activated FA concrete with a compressive strength of 58.2 MPa could be obtained using artificial aggregate as a substitute for natural granite and limestone aggregates. The mechanical properties of cement-activated FA aggregates were superior to those of alkali-activated FA aggregates. Qualitative and quantitative analyses revealed the formation mechanisms of artificial and natural aggregates in different matrices. Through continuous hydration and polymerization reactions, artificial aggregates gradually form narrower and denser interfacial transition zones with different matrices, especially in alkali-activated matrices. The continuously improved performance of the ITZ makes it less prone to forming cracks between the ITZ and the artificial aggregate. The weaknesses of artificial aggregate concrete exist not only in the ITZ but are also inherent in the aggregates themselves, resulting in lower mechanical properties and a large number of microcracks. Improving the mechanical properties of artificial aggregates can lead to the production of concrete with higher strength.
(2) The ITZs between cement paste and natural aggregates (e.g., granite and limestone) exhibited tight bonding. The elastic modulus at 28 d was >10 GPa, which increased slightly at 90 d. The ITZ width was 20–30 µm, and the principal hydration products were calcite crystals and C-S-H gel. The ITZ between cement paste and artificial aggregate at 28 d exhibited a relatively loose structure; its elastic modulus was more than 10 GPa and increased slightly at 90 d. The ITZ width was 30–40 µm at 28 d; it became narrower and denser at 90 d owing to ongoing chemical reactions, which improved the ITZ properties. The principal hydration products included C-S-H gel and calcite crystals.
(3) The ITZ between the alkali-activated paste and the limestone aggregate exhibited a dense structure. The elastic modulus of the ITZ at 28 d was 6.39 GPa, which increased rapidly to >10 GPa at 90 d. The ITZ width was 20–30 µm; N-A-S-H and C-A-S-H were the principal hydration products within the ITZ between the paste and the limestone aggregate. The ITZ between the alkali-activated paste and the artificial aggregate at 28 d exhibited a relatively loose structure with numerous pores; the elastic modulus was nearly 6.0 GPa and increased to more than 10 GPa at 90 d. The width of the ITZ was 30–40 µm at 28 d; the structures on both sides of the ITZ became more similar at 90 d, and the ITZ became much narrower and denser. The main difference in the ITZs formed by fly ash-based artificial aggregates in different matrices is that cracks are less likely to form in alkali-activated material matrices. Alkali-activated materials improved the properties of the ITZ and formed a denser, mechanically improved ITZ with fly ash-based artificial aggregates. The interfacial region exhibited significantly reduced porosity and improved micromechanical properties compared to natural aggregates, challenging the conventional view of the ITZ as inherently weaker in all cases.
(4) Several limitations of this study should be acknowledged. First, only one fly ash source was used; results may not generalize to all fly ash types. Second, the accelerated curing (75 °C, 100% RH) simulates precast production but not cast-in-place concrete. Third, the 1:1 aggregate-to-cementitious ratio was chosen for characterization purposes and differs from structural concrete proportions. Fourth, the maximum curing age was 90 days; longer-term behavior (>1 year) remains unknown. Fifth, SEM and nanoindentation alone cannot definitively prove complete ITZ disappearance—our conclusion is one of substantial densification and property convergence, not elimination. Finally, field performance under actual environmental and loading conditions was not evaluated. Future work should address these gaps. The use of fly ash-based artificial aggregates contributes to industrial waste valorization and reduces reliance on natural aggregate mining. Future work should also address durability (e.g., freeze–thaw, chloride ingress, and fire resistance), long-term performance under environmental loading, and a comprehensive life-cycle assessment (LCA) to quantify the environmental benefits of fly ash-based artificial aggregates relative to natural aggregates.

## Figures and Tables

**Table 1:** Chemical composition of cement and other raw materials (mass fraction, %).

Materials	SiO_2_	Al_2_O_3_	Fe_2_O_3_	CaO	MgO	Na_2_O	K_2_O	SO_3_
OPC	20.18	4.77	3.6	64.22	1.25	0.18	0.45	1.89
GGBS	34.14	14.2	1.0	40.14	7.54	0.42	0.41	0.07
FA	46.14	21.6	10.85	12.03	5.48	1.21	1.04	1.48

**Table 2:** Mixing ratios of cement–fly ash aggregate.

Sample No.	Cement/%	Fly Ash/%	W/C	Additives (SKY8588)/%
CFA	25	75	0.22	0.5

**Table 3:** Mixing ratios of alkali-activated fly ash aggregate.

Sample No.	Slag Powder/%	Fly Ash/%	W/C	Alkali Content/%	BaCl_2_/%
AAFA	20	80	0.30	6.0	0.5

**Table 4:** Mixing ratios of cement concrete without fine aggregate (kg/m^3^).

Sample No.	Cement	Aggregate	Water	Water–Cement Ratio
NFC-GA	950	950	360	0.38
NFC-LA	950	950	360	0.38
NFC-CA	950	690	360	0.38
NFC-AA	950	690	360	0.38

**Table 5:** Mixing ratios of alkali-activated concrete without fine aggregate (kg/m^3^).

Sample No.	Slag Powder	Fly Ash	Aggregate	Water	W/C	Alkali Content/%	Modulus/Ms
NFAAC-GA	190	760	950	285	0.30	6.0	1.3
NFAAC-LA	190	760	950	285	0.30	6.0	1.3
NFAAC-CA	190	760	690	285	0.30	6.0	1.3
NFAAC-AA	190	760	690	285	0.30	6.0	1.3

**Table 6:** Statistical analysis of the elastic modulus of ITZ for different types of concrete (GPa).

Sample No. (Refer to Table 4 and Table 5)	Age and Location					
	28 d			90 d		
	Matrix	ITZ	Aggregate	Matrix	ITZ	Aggregate
NFC-GA	22.05 ± 2.85	10.23 ± 2.59	54.66 ± 6.61	31.06 ± 9.25	10.58 ± 3.16	62.28 ± 5.75
NFC-CA	25.71 ± 5.48	11.32 ± 3.30	32.43 + 5.24	26.96 ± 7.60	12.09 ± 3.43	30.10 + 4.44
NFC-AA	26.13 ± 4.29	11.51 ± 1.92	38.60 ± 5.02	30.36 ± 7.16	11.81 ± 3.83	39.30 + 7.60
NFAAC-LA	20.16 ± 4.31	6.39 ± 0.79	58.68 ± 8.49	23.65 ± 6.67	12.02 ± 1.91	64.14 ± 8.69
NFAAC-CA	19.67 ± 3.94	5.58 ± 2.10	31.85 ± 5.69	21.91 ± 3.64	12.60 ± 2.42	35.88 ± 7.84
NFAAC-AA	20.46 ± 4.79	5.71 ± 2.43	31.25 ± 7.39	25.87 ± 4.02	10.61 ± 1.92	32.87 ± 1.79

## Data Availability

The original contributions presented in the study are included in the article, further inquiries can be directed to the corresponding authors.
